# Methodology for the development and calibration of the SCI-QOL item banks

**DOI:** 10.1179/2045772315Y.0000000034

**Published:** 2015-05

**Authors:** David S. Tulsky, Pamela A. Kisala, David Victorson, Seung W. Choi, Richard Gershon, Allen W. Heinemann, David Cella

**Affiliations:** 1 Department of Physical Therapy, University of Delaware, College of Health Sciences, Newark, DE, USA; 2 Kessler Foundation, West Orange, NJ, USA; 3 CTB/McGraw-Hill, Monterey, CA, USA; 4 Northwestern University Feinberg School of Medicine, Chicago, IL, USA; 5 Rehabilitation Institute of Chicago, Chicago, IL, USA

**Keywords:** Computer Adaptive Testing, Health-Related Quality of Life, Item Response Theory, Patient Reported Outcomes, Spinal Cord Injury

## Abstract

**Objective:**

To develop a comprehensive, psychometrically sound, and conceptually grounded patient reported outcomes (PRO) measurement system for individuals with spinal cord injury (SCI).

**Methods:**

Individual interviews (*n* = 44) and focus groups (*n* = 65 individuals with SCI and *n* = 42 SCI clinicians) were used to select key domains for inclusion and to develop PRO items. Verbatim items from other cutting-edge measurement systems (i.e. PROMIS, Neuro-QOL) were included to facilitate linkage and cross-population comparison. Items were field tested in a large sample of individuals with traumatic SCI (*n* = 877). Dimensionality was assessed with confirmatory factor analysis. Local item dependence and differential item functioning were assessed, and items were calibrated using the item response theory (IRT) graded response model. Finally, computer adaptive tests (CATs) and short forms were administered in a new sample (*n* = 245) to assess test-retest reliability and stability.

**Participants and Procedures:**

A calibration sample of 877 individuals with traumatic SCI across five SCI Model Systems sites and one Department of Veterans Affairs medical center completed SCI-QOL items in interview format.

**Results:**

We developed 14 unidimensional calibrated item banks and 3 calibrated scales across physical, emotional, and social health domains. When combined with the five Spinal Cord Injury – Functional Index physical function banks, the final SCI-QOL system consists of 22 IRT-calibrated item banks/scales. Item banks may be administered as CATs or short forms. Scales may be administered in a fixed-length format only.

**Conclusions:**

The SCI-QOL measurement system provides SCI researchers and clinicians with a comprehensive, relevant and psychometrically robust system for measurement of physical-medical, physical-functional, emotional, and social outcomes. All SCI-QOL instruments are freely available on Assessment Center^SM^.

## Introduction

The Spinal Cord Injury-Quality of Life (SCI-QOL) measurement system has been developed over the past 10 years to address the unmet need for comprehensive, conceptually relevant, psychometrically sound, and brief yet precise patient reported outcomes measures (PROs) for use in SCI research and practice. The end result of this work is a set of 19 item response theory (IRT)-calibrated item banks and 3 calibrated scales. Each item bank may be administered as a full bank, short form, or computer adaptive test (CAT), while scales may be administered in fixed-length format only. This manuscript outlines the methodologies used in the five phases of the SCI-QOL development project – namely, (1) subdomain selection, (2) item development, (3) field testing, (4) psychometric analysis and IRT calibration, and (5) testing in a new sample to assess of psychometric properties – and presents the results of graded response model item response theory calibration for each item bank.

## Background: 21^st^ century PRO measure development

Across all areas of health outcomes research, new standards have recently been introduced to guide the field of patient reported outcomes (PRO) measurement development efforts. Spearheaded by the Patient Reported Outcomes Measurement Information System (PROMIS), leading measurement experts who specialize in a wide variety of diseases and other health conditions have collaborated over the past 10 years to bring cutting-edge measurement techniques – most notably, those pioneered in the fields of educational and personality measurement – to health care. New Instrument Development and Scientific Standards^1^ documents have been developed to outline necessary steps in these development processes.

### PROMIS

Multiple federal initiatives have focused on developing health-related quality of life (HRQOL) measures for use in clinical trials. These efforts have focused on universally relevant measures that allow comparison of research findings across medical diseases and conditions. For instance, the National Institutes of Health (NIH) made the development of PROs part of their ‘roadmap’ for medical research in the 21^st^ century, a goal of which is to ‘catalyze changes necessary for transforming new scientific knowledge into tangible benefits for people.’^2^ The resulting Patient Reported Outcomes Measurement Information System (PROMIS)^3^ is a universally relevant measurement system with the potential for use in a wide variety of health care studies. PROMIS used state-of-the-art item writing and item pool development^4,5^ procedures that emphasized qualitative feedback and key stakeholder (e.g. patient) participation at multiple phases throughout the instrument development process. Stakeholder involvement helped guide the focus and development of the instrument, ensuring that the content of the resulting measures was conceptually grounded in phenomena deemed relevant and important from patients’ perspectives. Individuals with spinal cord injury (SCI) were included in this early development, making PROMIS one of the very few measurement systems to include people with SCI in the initial domain development.^6^ PROMIS is also unique from a methodological standpoint,^1,7^ in that advanced psychometric techniques^8^ were used to inform development of a computerized adaptive testing (CAT) platform for instrument administration.

### Neuro-QOL

In 2004, the National Institute of Neurological Disorders and Stroke (NINDS) prioritized the development of PROs as part of their efforts to develop common data elements for use in their research studies. Consequently, the Neurological Quality of Life (Neuro-QOL) measurement system was developed using the PROMIS Measurement Standards. The Neuro-QOL^9^ is a set of PRO item banks developed and validated for individuals with neurological disorders. In addition to adhering to the PROMIS development methodology, the Neuro-QOL incorporated many PROMIS items to facilitate linkage between the measurement systems. Neuro-QOL was designed for use with five neurological conditions: stroke, Parkinson's disease, multiple sclerosis, epilepsy, and amyotrophic lateral sclerosis. The measurement development process did not include individuals with SCI, and as such we found it necessary to develop a related measurement system with direct relevance and applicability to individuals with SCI. This effort is called the SCI-QOL measurement system.^10^

### SCI-QOL

SCI-QOL builds upon the foundation of clearly defined qualitative and quantitative methods and advanced psychometrics at the core of the PROMIS and Neuro-QOL systems. The research presented here describes the methods, measurement design, and results of the phases of this research project. A separate phase of research was conducted in regard to each of the project's specific aims, enumerated below, using a unique (a) sample, (b) set of scientific procedures, and (c) analytic methods. The methods included structured individual interviews with individuals with SCI, formal qualitative research using focus groups with individuals with SCI and clinicians, large-scale calibration field testing across multiple data collection sites, advanced psychometric analyses using IRT, and multisite testing of the newly developed SCI-QOL CAT and short form (SF) instruments.

**Figure 1:**
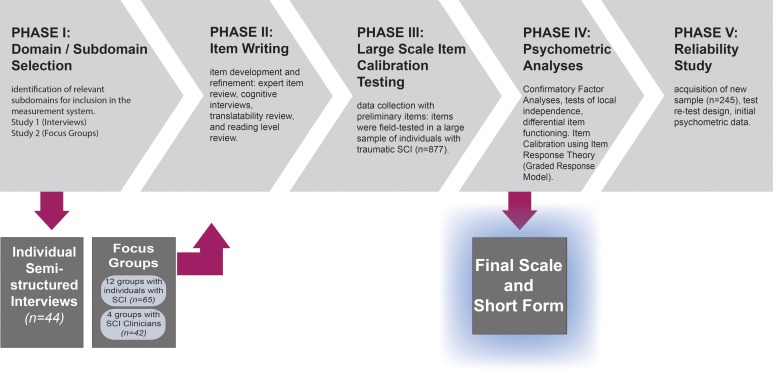
SCI-QOL Development and Calibration: Phases and Goals.

This manuscript provides a detailed description of the methods relevant to most of the manuscripts contained in this special issue. There were five primary aims to our development activities, which occurred and corresponded to five sequential phases of work. Our development goals included: (1) identification of relevant subdomains for inclusion in the measurement system (phase I studies: a – Individual Interviews and b – Focus Groups); (2) item development and refinement (phase II); (3) data collection with preliminary items (phase III); (4) psychometric analyses (factor analyses and item calibration; phase IV); and (5) acquisition of initial psychometric data (phase V). In this manuscript we report on the goals, methodology, and an overview of results in each of the five phases. A guide to the study phases and corresponding project aims is presented in Fig. 1. A glossary of terms is provided as Table 1.

**Table 1:** Glossary of Terms

Term	Description
Calibration	Using item response theory – in this case, graded response model analyses – to place all items on a single underlying metric so that any subset of items will yield a score that is directly comparable to the score on any other subset of items
Computer Adaptive Test (CAT)	‘Smart’ test that customizes administration for each individual. Each item that is administered is selected based on the participant's response to the previous item.
Construct	Conceptual area of interest/relevance; a construct may represent a domain, subdomain, or subtopic within a subdomain
Domain	Overarching, multidimensional conceptual area, e.g. emotional health, social participation, physical-medical health, physical function.
Instrument	Instruments are scales, assessment devices, or psychological tests. In this context, they are patient-reported outcomes measures.
Item Bank	Final set of items that have been calibrated with item response theory and are available for CAT administration
Item Pool	Set of preliminary items that have not yet been calibrated with item response theory
Measure	An assessment device or psychological test or scale. The terms ‘measure’ and ‘instrument’ refer to the same thing. In this context, we are referring to a patient reported outcomes scale and the ‘measure’ may be an item bank, short form, or CAT.
Metric	The underlying value that is used to understand the score and how it is scaled so that meaning can be derived from a score. For the SCI-QOL, the reported metric is a ‘T Metric’ with an average of 50 and standard deviation unit of 10. For PROMIS, the metric reflects the general population average. For SCI-QOL, the metric reflects either general population (when anchored to a PROMIS or Neuro-QOL scale) or to the SCI-population (when it is a new bank that does not have a comparable PROMIS or Neuro-QOL bank). The metric is relative the population that was used to calibrate the items
Phase	Major section of the original SCI-QOL development work with unique methods and goals. There are 5 distinct phases reported here.
Scale	A group of items measuring a similar construct. The scales indicate that the items are not administered via CAT technology but rather are a fixed-form set of items. These may or may not be calibrated using IRT.
Short Form	Brief (e.g. 6–10 items) fixed-length subset of items from a larger test or a calibrated item bank. When the short form is derived from a calibrated item bank, the short form score is directly comparable to the full bank or CAT score.
Subdomain	Subcomponent of a domain; unidimensional conceptual area that is amenable to item banking

## Phase I: Identification of SCI-QOL domains and subdomains

As described in Tulsky *et al.*,^10^ stakeholder feedback is imperative to ensure that the resultant measurement system is conceptually grounded to relevant HRQOL issues as actually experienced by individuals with SCI. We conducted two qualitative studies and consulted with individuals with SCI and other key stakeholders (i.e. SCI clinicians and researchers) to select the domains and specific subdomains to be developed into items banks as part of SCI-QOL measurement system.

### Study 1: Individual interviews

#### Study 1 (Individual interviews) - methods

We conducted IRB-approved semi-structured individual interviews with individuals with SCI to shape the conceptualization of HRQOL in SCI and to identify important themes and even items for inclusion in our new measure. These interviews focused on HRQOL among individuals with traumatic SCI (*n* = 44). Though largely a sample of convenience, participants were stratified to ensure representation of individuals with different diagnoses (tetraplegia vs. paraplegia), severity of injury (complete vs. incomplete), and time since injury (<1 year, 1–3 years, >3 years). Each interview consisted of open-ended questions about the general nature of HRQOL following injury and typically lasted about 2 hours. Participants were told that they should assume the role of expert, and were encouraged to raise issues resulting from their injuries. Interviewers recorded the responses verbatim and organized them by content area. These responses were used to identify thematic areas of HRQOL not captured in traditional HRQOL scales and to develop initial HRQOL items. Themes generated during Study 1 were used to inform the design and development of Study 2, and several verbatim quotes were used to form the basis of preliminary SCI-QOL items

#### Study 1 (Individual interviews) - results

Participant Demographic Characteristics. Forty-four community-dwelling individuals with traumatic SCI participated in the individual interviews. The mean age of participants was 42.6 years (SD 13.8). Seventy-three percent of participants were male, which is consistent with the demographic makeup of the overall SCI population (i.e. approximately 80% male).^11^ Fifty-five percent of the sample self-reported as Caucasian, 32% as Black or African-American, 7% Hispanic, and 7% Asian/Pacific Islander. The majority of participants (59%) were injured in automobile accidents. Fourteen percent of participants were injured by acts of violence, 12% of injuries were sports-related, 12% were sustained from falls, and 5% of injuries were due to other causes. Fourteen percent of the sample was diagnosed with complete tetraplegia, 34% with incomplete tetraplegia, 30% with complete paraplegia, and 23% with incomplete paraplegia. On average, participants were 8.0 years post injury (SD 11.0), with 27% less than one year post injury, 32% between 1–3 years post injury, and 39% were greater than 3 years post injury.

Preliminary HRQOL Domains*.* Based on direct quotes from participants, study team members drafted a total of 1,289 preliminary HRQOL items across Emotional, Physical-Medical, Physical-Functional, Social/Participation, Environmental Factors, and Sexual Functioning domains. Table 2 provides counts of preliminary items by domain and subdomain.

**Table 2:** Preliminary Items Generated from Study 1: Item Generation Interviews

Domain	Subdomain	*n*	Overall Percent	Percent within Domain
Emotional	Attitude	118	9.2	33.3
	Body image	10	0.8	2.8
	Coping	47	3.6	13.3
	Emotional well-being	92	7.1	26.0
	Life satisfaction	51	4.0	14.4
	Spirituality	36	2.8	10.2
Environmental Factors	Assistive devices	51	4.0	16.2
	Accessibility/environmental	82	6.4	26.1
	Finances	65	5.0	20.7
	Housing	18	1.4	5.7
	Home health attendant	34	2.6	10.8
	Insurance	14	1.1	4.5
	Transportation	50	3.9	15.9
Other	Other	31	2.4	100.0
Physical-Functional	Physical well-being	120	9.3	100.0
Physical-Medical	Aging with SCI	4	0.3	5.8
	Bowel/Bladder management	37	2.9	53.6
	Pain	28	2.2	40.6
Sexual Functioning	Sexuality	43	3.3	100.0
Social/Participation	Able-bodied persons	59	4.6	16.5
	Communication	22	1.7	6.2
	Education	18	1.4	5.0
	Employment (or school)	38	2.9	10.6
	Future directions	4	0.3	1.1
	Independence	71	5.5	19.8
	Leisure or Hobby	20	1.6	5.6
	Relationships – family	28	2.2	7.8
	Relationships – Parenting	8	0.6	2.2
	Relationships – Professional	9	0.7	2.5
	Relationships – Romantic	5	0.4	1.4
	Relationships – Social/Friends	51	4.0	14.3
	Support	25	1.9	7.0
		1289	100.0	

### Study 2: Focus groups

#### Study 2 (Focus groups) - methods

In our second qualitative study, we conducted formal, semi-structured focus groups to identify key domains and subdomains of HRQOL^12,13^ and to inform the selection and/or development of individual HRQOL items. Participants included individuals with SCI and SCI clinicians who were recruited through four National Institute on Disability and Rehabilitation Research (NIDRR) – funded Model SCI System sites.^12,14^ Individuals with SCI were included in this study if they had sustained a traumatic SCI and were able to read and understand English. Each site made efforts to recruit individuals with SCI across all levels of injury, and to include representatives from various racial, ethnic, gender, and socioeconomic groups to help ensure a heterogeneous makeup of the overall sample. The SCI professional groups included physiatrists, physical therapists, psychologists, and nurses who work primarily (i.e. ≥50% of the time for a minimum of 3 years) with persons with SCI.

The focus group moderators were the study PI (author DST) and a Ph.D.-level co-investigator (author DV) who had extensive experience (i.e. >20 groups) conducting focus groups related to HRQOL and measurement development. The co-moderators debriefed (i.e. discussed things that went well and things that could be improved for future groups) following each focus group session to help ensure adherence to the focus group guide and method for the remaining groups. Moderators facilitated discussions in a semi-structured manner, providing basic rules and general topics for discussion yet allowing participants to discuss their own stories and perspectives. Participants were encouraged to discuss experiences and issues that affected their QOL and moderators prompted participants to focus on both positive and negative aspects of life with SCI in order to ensure a range of feedback.

After discussing experiences with their own QOL or, in the case of clinicians, the experiences of their patients, focus group participants were asked to define QOL and outline what they perceived to be the most important aspects of QOL for an individual with SCI. Following this general discussion, different patient groups were asked to focus on one specific domain area, e.g. physical health, emotional health, or social participation. A parallel set of focus groups covered physical functioning and activity limitations; the results from these focus groups are reported elsewhere.^14,15^ Each professional group covered all of the above domains of functioning. Group discussions were audio-recorded and transcribed verbatim.

We used a grounded-theory^16,17^ based qualitative approach, as described in Kisala and Tulsky,^13^ to analyze focus group data. A minimum of two investigators reviewed each transcript independently. Analysis steps included independent transcript review (open coding), development of a hierarchical (axial coding), and application of codes to each segment of transcript text (selective coding) by two independent raters. Raters logged and reconciled disagreements in order to achieve 100% agreement. This final code for each chunk of text was used to calculate the relative frequency of mention for various focus group topics. A detailed description of qualitative analysis results may be found in Tulsky *et al.*^12^

#### Study 2 (Focus groups) - results

A total of 16 focus groups were held across the 4 sites, as follows: 12 groups with individuals with SCI (*n* = 65) and 4 groups with SCI clinicians (*n* = 42). The focus group results, participant demographic characteristics, and a more thorough description of their methodology have been published.^12,13^ Both individuals with SCI and clinicians who work with people with SCI focused on similar issues during the focus group discussions, nominating a variety of subdomains across the larger domains of physical-medical, emotional and social functioning.

### Literature review

To be as comprehensive as possible in the selection of subdomains for inclusion, the research team reviewed the literature on each subdomain within the context of SCI, identified key component issues and symptomatology, and identified extant scales where applicable. In domains that overlapped conceptually with PROMIS and Neuro-QOL item banks, the research team used the literature searches conducted by the PROMIS^3^ and Neuro-QOL^9^ study teams

### Additional stakeholder input

Informal interviews with clinicians and researchers with expertise in SCI medicine helped guide the domain selection. Additionally, we held a series of interactive discussions with a regional SCI consumer advisory board that provided input on study methodology and interim results (e.g. preliminary lists of subdomains for inclusion). The advisory board met quarterly and consisted of individuals with SCI, many of whom worked in the area of disability services and/or held leadership positions within the disability community. This advisory board reviewed project progress and provided input on the applicability of each component of the proposed measurement system.

### Final subdomain selection

Taking into consideration all of the input described thus far, the SCI-QOL project team selected 30 subdomains for further development (Table 3). These included 7 item pools related to physical-medical issues, 8 item pools related to emotional functioning, 6 item pools related to participation and social functioning, and 4 item pools related to sexual functioning. The development of the 5 ‘Spinal Cord Injury-Functional Index (SCI-FI)’ item pools related to physical functioning has been described previously,^14,18^ therefore these pools are not included among those described here.

**Table 3:** Subdomains for development, field testing, and analysis

Domain	Developed	Tested	Analyzed
Emotional Health			
Positive Affect & Well-Being	X	X	X
Depression	X	X	X
Anxiety	X	X	X
Stigma	X	X	X
Resilience	X	X	X
Grief/Loss	X	X	X
Self-Evaluation	X	X	X
Psychological Trauma	X	X	X
Physical-Medical Health			
Pressure Ulcers	X	X	X
Bladder Management Difficulties	X	X	X
Bladder Complications	X	X	X
Bowel Management Difficulties	X	X	X
Pain Interference	X	X	X
Pain Behavior	X	X	X
Respiratory Functioning	X	X	
Social Participation			
Ability to Participate	X	X	X
Satisfaction with Social Roles & Activities	X	X	X
Independence	X	X	X
Control over Participation	X	X	X
Involvement in Life Situations	X	X	X
Environmental Barriers & Supports	X		
Physical Functioning			
Ambulation	X	X	X
Basic Mobility	X	X	X
Fine Motor Function	X	X	X
Self Care	X	X	X
Wheelchair Mobility	X	X	X
Sexual Functioning			
Sexual Function–Performance	X	X	
Sexual Function–Satisfaction	X	X	
Sexual Self Esteem	X	X	
SCI Interfering Factors	X	X	

## Phase II: item development and refinement

Once it was determined which subdomains would be selected for further development, the goal of the second phase of the study was to develop and refine component items in each topic area. The comments made during the individual interviews and focus groups not only guided selection of relevant domains for inclusion but also contributed directly to the item pools, with participant quotes forming the initial basis of many included items. As described by Kisala and Tulsky,^13^ the initial step in this process is to define the topic of interest and the scope of the ultimate scale.^10,12^ Since the goal was to develop an IRT-based scale, we needed to select or write sets of items that address a single underlying construct, with items at various levels of ‘difficulty’ arranged across the construct being measured. For each subdomain, a preliminary item pool was developed using the qualitative feedback from the individual interviews and focus groups to prepare item stems that employed wording used by participants. In subdomains with content overlap with existing measures, extant items were also included, especially items from the Neuro-QOL and PROMIS scales. By using items from PROMIS and Neuro-QOL verbatim, the scores on the SCI-QOL and PROMIS (and SCI-QOL and Neuro-QOL) could be calibrated using IRT-based linking methods to obtain a common metric between the tests. In other words, the common items between the SCI-QOL and PROMIS (or SCI-QOL and Neuro-QOL) serve as anchor items, and through IRT-based linking methods, we can transform SCI-QOL item parameter estimates to the PROMIS or Neuro-QOL metric, enabling direct comparisons with these other PRO measurement scales where relevant. For linkage, only a substantial number of common items need to be used and additional items could be included to ensure content coverage in one of the populations. For SCI-QOL, additional items were written for any relevant area of functioning that was discussed in the SCI medical literature even if it was not described in detail by participants in the qualitative studies.

### Qualitative item review process

Each item pool underwent qualitative item review (QIR) to optimize the content, wording, and construct coverage of the included items. The first step in this process involved iterative expert reviews in which members of the investigative team reviewed all items for relevance, redundancy, and wording. Poorly worded items or items that reflected multiple concepts were identified in a team meeting with investigators and reworded. The investigators reviewed each set of items to ensure that they appeared to be related to a similar construct, and also organized the items along a hierarchy of difficulty. Items flagged as not construct representative were removed, gaps in the difficulty continuum were identified, and new items were written to help bridge any gaps in the continuum.

Next, a series of cognitive debriefing interviews were held with individuals with SCI (*n* = 5 per item) who, in a structured interview format, read each item, responded based on their level of functioning, and then reviewed the meaning of the item and the cognitive processes that led to their response. Participants were asked to discuss the relevance and wording of each item and to identify items that were vague or ambiguous. This process helped ensure that the items selected for testing would be understood as intended by respondents.

Next, we evaluated each item to make sure it would be comprehensible to all participants capable of reading English, regardless of education level. The Lexile Framework™ ^19^ was used to ensure that all items were worded at or below a 5^th^ grade reading level.

For the final QIR step, we conducted a translatability and cultural review^20^ of all newly developed items to ensure that item wording would not preclude translation to Spanish at a later time. A team of translation science experts reviewed each item, identifying specific words or item content that would be difficult to translate or would be culturally inappropriate for Spanish-speaking individuals with SCI, and suggested different ways of stating the item in order to make the final scale more universally relevant for future translation into Spanish.

All item pools were finalized except for one related to environmental factors (i.e. barriers and facilitators to participation such as economic factors). This item pool was removed from the SCI-QOL and, to optimize resource allocation, migrated to a concurrent project established to develop a measure of environmental factors that impede or enhance social participation in individuals with disabilities.^21^

Several of the finalized item banks were unique and new and based upon the SCI qualitative feedback and literature. The domains that they measured had not been covered by existing Neuro-QOL or PROMIS item banks. However, there were also issues and domains that were more universal, experienced by the general population as well as the SCI population. In these instances, SCI-QOL item banks cover subdomains that had already been measured by existing PROMIS or Neuro-QOL item banks (e.g. Depression, Pain Interference, Ability to Participate in Social Roles). In these cases, the SCI-QOL ‘version’ of each item bank is based on the original PROMIS or Neuro-QOL bank through the use of common, verbatim items. However, the new samples of individuals with SCI collected as part of this study were used to re-calibrate these item banks in an SCI sample so that the items banks would be optimized for use in individuals with SCI. To ensure comparability and interpretability with the PROMIS and Neuro-QOL scales, the item calibration parameters were transformed to the PROMIS (or Neuro-QOL) metric. To ensure that sufficient common items were available to link, 182 items from Neuro-QOL and 56 items from PROMIS (22 of those items were common to Neuro-QOL and PROMIS) were merged back into the pool along with the 510 new items. The final set of SCI-QOL items totaled 726 items across 24 pools, covering the various domains and sub-domains of Physical Medical, Emotional, Social Health, and Sexual Functioning. Additionally, 324 items in a fifth domain area (physical function) were developed and tested in a companion project, the Spinal Cord Injury – Functional Index (SCI-FI).^14,18^

## Phase III: Field testing

We calibrated the item pools using an IRT Graded Response Model (GRM).^22^ Estimation of the GRM requires a participant sample that is heterogeneous with regard to functioning (i.e. representative of the population) and large enough (e.g. n ≥ 500) to produce stable parameter estimates.^23^

### Phase 3: field testing - Participants

A sample of 877 individuals with SCI was recruited from five SCI Model Systems (SCIMS) centers and SCI Center of Excellence in the Department of Veterans Affairs: University of Michigan, Kessler Foundation/Kessler Institute for Rehabilitation, Rehabilitation Institute of Chicago, University of Washington, Craig Hospital, and the James J. Peters/Bronx VA Medical Center. The study protocol was reviewed and approved by each site's Institutional Review Board. Persons with a documented traumatic SCI who were 18 ys. or older and could read, speak, and understand English fluently were eligible to participate. Recruitment goals were stratified by diagnosis (paraplegia vs. tetraplegia), completeness of injury (complete vs. incomplete), and time since injury (<1 year, 1–3 years, and >3 years) to ensure that the final sample was heterogeneous with regard to SCI-specific characteristics. Each participant's diagnosis was confirmed by medical records, and each participant's neurologic level was documented by their most recent American Spinal Injury Association Impairment Scale (AIS) rating.^24^

### Phase 3: field testing - Data collection procedures

All items were presented in a structured interview to participants either in person or over the phone. Each interviewer received in-person training in interviewing techniques and used a semi-structured script to ensure standardization of the interview format. A detailed Manual of Procedures was prepared and distributed to all sites. Throughout the data collection for this calibration study, interviewers from all sites participated in biweekly conference calls with the study coordinator to discuss progress and goals, specifically with regard to meeting sampling stratification goals.

Due to the large number of items in the calibration version of the SCI-QOL study (*k* = 726), data collection was divided into three sessions. An additional interview session was held to administer the physical functioning item pools, and many of the same participants elected to participate in that session. All items within an individual item pool (e.g. Pain Interference) were administered during the same session. All responses were entered into a customized web-based data collection software system that allowed data to be automatically uploaded and stored on a secure server immediately. Because the response options differed somewhat from one set of items to the next, participants were shown a response card to facilitate their responses.

### Phase 3: field testing - Participant demographic characteristics

Of the total sample of 877 individuals, 757 completed Session 1 (containing items related to Physical Medical issues), 717 completed Session 2 (Emotional Well-Being items), and 641 completed Session 3 (Social Participation, Stigma, and Sexual Functioning items). While each participant was encouraged to complete all of the sessions, this was not required and therefore different sample sizes were obtained for each interview session.

Among the 877 total participants, mean age was 42.9 years (SD 15.4) and 79% of participants were male. Of the sample, 70% self-identified as Caucasian, 18% as African-American, 2% as more than one race, 2% as Asian, and 8% as Other. Additionally, 11% of participants were of Hispanic or Latino origin or descent. In terms of level and completeness of injury, 24% of participants were diagnosed with complete paraplegia, 18% with incomplete paraplegia, 23% with complete tetraplegia, and 34% with incomplete tetraplegia. The average time since injury was 6.7 years (SD = 9.9): 28% of participants had been injured for less than 1 year at the time of study participation, 27% between 1 and 3 years, and 45% for more than three years. More detailed demographic information for the overall sample, as well as sample demographics for the individual sessions, may be found in Table 4.

**Table 4:** Calibration Sample Demographics

Variable	Total Sample	Session 1 (Physical)	Session 2 (Emotional)	Session 3 (Social)
	(*n* = 877)	(*n* = 757)	(*n* = 717)	(*n* = 641)
Age	42.9 years (SD 15.4)	42.9 years (SD 15.5)	43.0 years (SD 15.3)	42.9 years (SD 15.3)
Age at Injury	36.3 years (SD 16.6)	36.3 years (SD 15.8)	36.1 years (SD 16.8)	35.9 years (SD 16.9)
Sex				
Male	693 (79%)	599 (79%)	559 (78%)	496 (77%)
Female	184 (21%)	158 (21%)	158 (22%)	145 (23%)
Ethnicity				
Hispanic	93 (11%)	80 (11%)	82 (11%)	66 (10%)
Non-Hispanic	771 (88%)	665 (88%)	631 (88%)	571 (89%)
Not provided	13 (2%)	12 (2%)	4 (1%)	4 (1%)
Race				
Caucasian	613 (70%)	536 (71%)	505 (70%)	460 (72%)
Black or African-American	155 (18%)	130 (17%)	125 (17%)	110 (17%)
Asian	13 (2%)	11 (2%)	8 (1%)	6 (1%)
American Indian/Alaska Native or Native Hawaiian/Pacific Islander	8 (1%)	7 (1%)	7 (1%)	5 (1%)
More than one race	13 (2%)	11 (2%)	9 (1%)	7 (1%)
Other	60 (7%)	51 (7%)	50 (7%)	42 (7%)
Not provided	15 (2%)	11 (2%)	13 (2%)	10 (2%)
Education				
High school or less	338 (39%)	290 (38%)	275 (38%)	241 (38%)
Some college	297 (34%)	253 (34%)	248 (35%)	217 (34%)
Bachelor's degree or more	240 (27%)	212 (28%)	194 (27%)	183 (29%)
Total Household Income				
<$20,000	223 (25%)	184 (24%)	193 (27%)	169 (26%)
$20,000–74,999	306 (35%)	269 (36%)	268 (37%)	233 (36%)
>$75,000	195 (22%)	167 (22%)	148 (21%)	144 (22%)
Unknown/Not provided	153 (17%)	137 (18%)	108 (15%)	95 (15%)
Time Since Injury				
<1 year post injury	246 (28%)	218 (29%)	196 (27%)	139 (22%)
1–3 years post injury	239 (27%)	210 (28%)	186 (26%)	192 (30%)
>3 years post injury	392 (45%)	329 (44%)	335 (47%)	310 (48%)
Diagnosis				
Paraplegia Complete	200 (23%)	173 (23%)	171 (24%)	151 (24%)
Paraplegia Incomplete	152 (17%)	134 (18%)	136 (19%)	116 (18%)
Tetraplegia Complete	193 (22%)	167 (22%)	152 (21%)	129 (20%)
Tetraplegia Incomplete	284 (32%)	249 (33%)	225 (31%)	215 (34%)
Not confirmed*	48 (6%)	34 (5%)	31 (4%)	27 (4%)
Wheelchair Use *(not mutually exclusive)*				
Manual Wheelchair	462 (53%)	386 (61%)	386 (54%)	342 (53%)
Power Wheelchair	367 (42%)	313 (41%)	305 (43%)	259 (40%)
Cause of Injury				
Motor vehicle accident	294 (34%)	245 (32%)	241 (34%)	219 (34%)
Gunshot wound or other violence	105 (12%)	89 (12%)	83 (12%)	77 (12%)
Fall	193 (22%)	169 (22%)	164 (23%)	140 (22%)
Dirt bike accident	7 (1%)	6 (1%)	6 (1%)	7 (1%)
Motorcycle accident	23 (3%)	20 (3%)	20 (3%)	16 (3%)
Bicycle accident	10 (1%)	9 (1%)	9 (1%)	8 (1%)
Other Sports	58 (7%)	56 (7%)	45 (6%)	47 (7%)
Diving	62 (7%)	50 (7%)	57 (8%)	49 (8%)
ATV accident	8 (1%)	4 (1%)	4 (1%)	6 (1%)
Medical or surgical accident	29 (3%)	28 (4%)	27 (4%)	18 (3%)
Other	51 (6%)	47 (6%)	49 (7%)	41 (6%)
Not reported	37 (4%)	81 (11%)	12 (2%)	13 (2%)

Note: Participant demographic information on the physical functioning sample may be found in Tulsky *et al*.^12^ and Jette *et al*.^16^ ^*^For the small percentage of individuals without confirmed medical record information, self-reported diagnosis and completeness were used throughout the analyses.

## Phase IV: Psychometric analysis

The analysis steps used in this project followed closely in line with those outlined by Reeve *et al.*,^25^ including evaluation of dimensionality and estimation of IRT-based item parameters. We examined the dimensional structure of each item pool, ensuring that each represented an essentially unidimensional construct which is a prerequisite for conducting IRT analysis. We used the results to evaluate the appropriateness of the SCI-QOL items in each pool, and to inform the removal of biased or misfitting items from each final item bank. The final goal of this phase was to obtain IRT parameters of the items to develop a computer adaptive test (CAT) and provide data for use in short form item selection.

We evaluated internal consistency (Cronbach's alpha), corrected item-total correlations, data completeness, and underlying dimensionality of responses. Since unidimensionality is a prerequisite for conventional IRT analysis and CAT, dimensionality of each bank was assessed using confirmatory and exploratory factor analyses (CFA/EFA). We tested all items in the bank on their fit to a unidimensional construct. Several indices of goodness-of-fit served as criteria for acceptable unidimensionality, including Bentler's Comparative Fit Index (CFI),^26^ the Tucker-Lewis Index (TLI)^27^ (where values of 0.90 or above indicate acceptable fit to the model and values of 0.95 or above indicate good fit),^28^ and the root mean square error of approximation (RMSEA)^29^ (where values below 0.08 indicate acceptable fit, and values below 0.06 are considered good fit). We assessed local item dependence (LID), which occurs when a pair of items violates the underlying assumption that responses to individual items should be uncorrelated with each other at any given level of the construct being measured. A criterion of residual correlation >0.2 was used to identify item pairs with potentially problematic LID. Unidimensional models were tested in separate CFA analyses for each of the 20 item pools. When poor fit to a unidimensional model was indicated by model fit statistics (e.g. CFI, TLI, RMSEA), we examined the entire item bank and selected items for removal based on low factor loadings or the presence of LID. Analyses were then iteratively re-run after each wave of item removal until CFA results supported a unidimensional model, all items exhibited satisfactory factor loadings (i.e. ≥0.30; ideally ≥0.40), and LID was minimized.

IRT parameters and IRT-based model fit were subsequently estimated using the Graded Response Model. Within each item bank, each item was evaluated for misfit (S-X^2^ index)^30^ and differential item functioning (DIF)^31^ for age, sex, education level, diagnosis (paraplegia vs. tetraplegia), completeness of injury (complete vs. incomplete), and time since injury (<1 year vs. >1 year). If any additional items were removed from the item pool at this time, the IRT and DIF analyses were re-run following their removal.

Next, item banks containing a substantial number of items from PROMIS (e.g. Anxiety, Depression, Pain Interference) or Neuro-QOL (e.g. Positive Affect and Well-Being, Ability to Participate in Social Roles and Activities, Satisfaction with Social Roles and Activities) were transformed onto the PROMIS or Neuro-QOL metric as appropriate, using IRT-linking techniques. This linking procedure utilized common items as ‘anchors,’ using Stocking-Lord^32^ linking techniques^33^ to identify slope and intercept transformation constants, and performing a linear transformation of each item calibration so that SCI-QOL item parameters were placed on the respective PROMIS or Neuro-QOL metric. In other words, the item parameters were estimated based on SCI samples in order to obtain most optimal (reliable and valid) estimates and then transformed to the PROMIS/Neuro-QOL metric to facilitate comparisons across populations. This procedure provides the dual advantage of having a SCI-specific sample inform the optimal CAT item selection algorithm, thereby ensuring the administration of the most informative item at each level of the underlying trait, while still allowing direct comparison with the general population^34^ and/or other studies via the respective PROMIS or Neuro-QOL metric. For subdomains that did not have a comparable PROMIS or Neuro-QOL item bank (i.e. an item bank that was unique to SCI), the calibrations based upon the SCI sample were used to develop the IRT parameters.

### Phase 4: Psychometric analysis - CAT programming

CAT is a dynamic way to present a select subset of items from a calibrated item bank that are specifically relevant to the individual being assessed. For example, on the Depression CAT, someone who indicates that they ‘Never’ feel sad will not see items about suicidal ideation. As described by Cella *et al.*, IRT-calibrated items are considered ‘pre-validated’ (pg. 134),^35^ wherein the score on any subset of these items, whether administered by CAT or as a static short form (SF), is directly comparable to the full-bank score.

For this project, once all analyses were completed and all parameters transformed (where applicable), our final step was to develop CATs and static short forms (SF) for each item bank. The CATs were developed using final (transformed) item calibration parameters obtained from the last iteration of the IRT analyses. The final IRT parameters were programmed into the Assessment Center^SM^ platform,^36^ available at http://www.assessmentcenter.net. SCI-QOL uses the default PROMIS CAT settings so that a minimum of 4 items are administered to each person. The CAT then continues to administer items until the standard error of measurement falls below 0.3 or until a maximum of 12 items is administered. This typically results in a CAT length of 6–8 items For most item banks, Assessment Center may also be set to administer any item on the SCI-QOL short form that has not been selected by the CAT so that both Short Form and CAT scores can be obtained.

### Phase 4: Psychometric analysis - Short form selection

A short form (SF) is a brief, fixed-length version of an item bank that is developed using a balance of psychometric and clinical considerations. Since all items in each bank are calibrated on a single underlying metric, the score on a given SF is directly comparable to the CAT or full bank administration of the same bank. For each item bank, a small group of co-investigators, including at least one individual with clinical/topic-area expertise and two measurement experts, reviewed the difficulty (location) and slope (discrimination) parameters for each item. As a starting place, items were divided into quintiles based on location (i.e. the mean of category threshold parameters for each item), and at each quintile, the 1–2 item(s) with the highest slope were chosen. Clinical relevance, item wording, and, in an effort to include a diverse set of items on each form, similarity to other included items were also considered. All SFs contain between 7 and 10 items and are available through the Assessment Center or from the corresponding author. Bivariate correlations (Pearson's *r*) between CAT and SF scores on each item bank were computed using Firestar^37^ CAT simulations with calibration data. For our a priori hypothesis, we expected high correlations (i.e. approaching 1.0) between different modes of administration (i.e. CAT versus short form) of the same SCI-QOL item bank.

### Phase 4: Psychometric analysis - Scoring metric for SCI-QOL item banks

All SCI-QOL scores have been transformed to a T-metric, with a mean of 50 and standard deviation of 10. For all banks that have been linked and placed on the PROMIS or Neuro-QOL metric (see Table 5), the population used to calibrate the extant item bank serves as the reference group.

**Table 5:** Linkages with PROMIS and Neuro-QOL

Subdomain/Bank	# SCI-QOL Items	Linked to	# PROMIS Items	# Neuro-QOL Items	Reference Population
**Bladder Management Difficulties**	15	–	0	0	SCI
**Bladder Complications**	5	–	0	0	SCI
**Bowel Management Difficulties**	26	–	0	0	SCI
**Pressure Ulcers**	14	–	0	0	SCI
**Pain Interference**	7	PROMIS	**18**	0	General
**Pain Behavior**	4	PROMIS	**3**	0	General
**Positive Affect & Well-being**	5	Neuro-QOL	0	**14**	General
**Depression**	6	PROMIS	**18**	22	General
**Anxiety**	2	PROMIS	**18**	5	General
**Stigma**	5	Neuro-QOL	0	**12**	Neuro
**Resilience**	21	–	0	0	SCI
**Grief/Loss**	17	–	0	0	SCI
**Self-Evaluation**	19	–	0	3	SCI
**Psychological Trauma**	19	–	0	0	SCI
**Ability to Participate in Social Roles & Activities**	0	Neuro-QOL	0	**19**	General
**Satisfaction with Social Roles & Activities**	2	Neuro-QOL	3	**14**	General + Neuro
**Independence/Autonomy**	8	–	0	0	SCI
**Ambulation**	39	–	4	9	SCI
**Basic Mobility**	54	–	6	9	SCI
**Fine Motor Function**	36	–	9	6	SCI
**Self Care**	90	–	11	10	SCI
**Wheelchair Mobility**	56	–	0	0	SCI

Note: **Bold text** indicates number of items that are statistically linked.

Abbreviations: Neuro, neurological population consisting of individuals with stroke, epilepsy, Parkinson's, multiple sclerosis, and amyotrophic lateral sclerosis.

All PROMIS v1.0 item banks and the majority of Neuro-QOL banks were developed using general population samples. In these cases, the person's score on the SCI-QOL reflects their standing in the general population. An exception to this is Stigma, where scores on this bank reflect individuals' standing in reference to a mixed neurological sample (i.e. stroke, epilepsy, Parkinson's disease, amyotrophic lateral sclerosis). For all banks that are ‘new’ to SCI-QOL (e.g. Bladder Management, Bowel Management, Resilience, Grief/Loss), an individual's score represents their standing among individuals with traumatic SCI.

### Phase 4: Psychometric analysis - Scoring direction

In keeping with PROMIS convention, higher scores on an instrument indicate more of the trait being measured. As seen in Table 6, this means that SCI-QOL item banks/scales that measure positive constructs (e.g. Resilience, Positive Affect and Well-Being) are scored in a positive direction, with higher scores indicating better functioning. Item banks/scales that measure a negative construct (e.g. Depression, Bladder Management Difficulties) are scored in a negative direction, with higher scores indicating greater symptom severity.

**Table 6:** SCI-QOL Item Bank Statistics

Domain	Bank	Scoring Direction	No. Items Tested	No. Final Items	No. Items in SF	N	CFI	RMSEA	α	Item Total Correlations	Slope Range	Threshold Range
Physical Health	Pain Interference	Severe Symptom	28	25	10	757	0.983	0.063	0.968	0.47 to 0.86	1.22 to 4.27	0.00 to 2.66
	Pain Behavior	Severe Symptom	18	7	–	757	0.996	0.076	0.899	0.59 to 0.81	2.26 to 5.39	−0.62 to 2.31
	Bladder Mgmt. Difficulties	Severe Symptom	38	15	8	757	0.965	0.093	0.905	0.38 to 0.78	1.05 to 4.21	0.10 to 3.23
	Bowel Mgmt. Difficulties	Severe Symptom	53	26	9	757	0.955	0.078	0.951	0.32 to 0.79	0.92 to 4.90	−0.01 to 3.68
	Pressure Ulcers	Severe Symptom	30	14^†^	7	189	0.961	0.124	0.924	0.56 to 0.80	2.17	−0.85 to 2.02
	Bladder Complications	Severe Symptom	8	5^†^	–	297	0.982	0.080	0.794	0.28 to 0.68	1.51	−0.33 to 3.34
Emotional Health	Positive Affect & Well-being	Better Function	32	28	10	717	0.947	0.094	0.970	0.61 to 0.82	2.25 to 4.55	−2.19 to 1.79
	Depression	Severe Symptom	35	28	10	716	0.968	0.066	0.964	0.51 to 0.81	1.39 to 4.23	−0.68 to 3.14
	Anxiety	Severe Symptom	38	25	9	716	0.953	0.069	0.946	0.50 to 0.74	1.49 to 2.95	−0.91 to 3.38
	Stigma	Severe Symptom	30	23	10	611	0.941	0.088	0.936	0.39 to 0.72	1.81 to 4.72	−0.17 to 2.77
	Resilience	Better Function	32	21	8	717	0.968	0.074	0.951	0.54 to 0.78	1.48 to 3.26	−3.26 to 1.11
	Grief/Loss	Severe Symptom	20	17	9	716	0.976	0.078	0.947	0.59 to 0.78	1.65 to 3.15	−1.48 to 2.48
	Self-Esteem	Better Function	30	23	8	716	0.946	0.087	0.950	0.50 to 0.81	1.28 to 3.74	−3.94 to 1.38
	Psychological Trauma	Severe Symptom	31	19	8	716	0.954	0.065	0.903	0.45 to 0.69	1.17 to 2.52	−0.78 to 3.50
Social Participation	Ability to Participate in SRA	Better Function	50	27	10	641	0.946	0.096	0.963	0.51 to 0.81	2.11 to 5.66	−2.08 to 0.25
	Satisfaction with SRA	Better Function	50	35	10	641	0.914	0.093	0.971	0.50 to 0.77	2.63 to 5.84	−2.06 to 0.07
	Independence	Better Function	13	8	8	641	0.980	0.111	0.894	0.55 to 0.79	1.54 to 3.74	−2.59 to 1.05
Physical Functioning	Ambulation	Better Function	40	39	11	855	0.999	0.039	0.991	0.45 to 0.94	3.23 to 7.29	0.48 to 3.35
	Basic Mobility	Better Function	65	54	11	855	0.969	0.081	0.985	0.54 to 0.90	1.19 to 7.84	−2.15 to 1.73
	Fine Motor Function	Better Function	39	36	9	850	0.998	0.049	0.990	0.58 to 0.94	1.95 to 6.96	−1.82 to 1.05
	Self Care*	Better Function	99	90	11	850	0.992	0.052	0.995	0.21 to 0.92	0.90 to 5.73	−5.05 to 0.75
	Wheelchair Mobility	Better Function	63	56	10^a^/9^b^	709	0.929	0.063	0.993	0.29 to 0.82	0.90 to 4.31	−3.79 to 1.64

^a^Number of items in Manual Wheelchair Short Form.

^b^Number of items in Power Wheelchair Short Form.

*For Males, 85 items are included in Self-Care, CFI is .992, and RMSEA is 0.049. For Females, 84 items are included, CFI is 0.993, and RMSEA is 0.049.

^†^The Pressure Ulcers and Bladder Complications scales each include one additional non-scored screener item.

## Phase IV: Psychometric analysis - RESULTS

Psychometric analyses were conducted on 17 of the 24 tested item pools (see Table 3 for a list of analyzed pools). Of the 17 analyzed item pools, 14 calibrated item banks and 3 brief, fixed-length scales were developed. See Table 7 for a brief domain definition for each final bank/scale. Additionally, expanded definitions may be found in Tulsky *et al.*^10^ in this issue for the domain definitions for final banks/scales.

**Table 7:** SCI-QOL Domain Definitions

Physical Health
Bladder Management Difficulties
A range of difficulties associated with bladder management, ranging from ability to carry out their bladder program to worry about bladder accidents, performing their bladder program, and impact on everyday living
Bladder Complications
A range of difficulties associated with bladder complications, such as urinary tract infection (UTI), UTI impact on everyday living, and bladder issues affecting sexual function
Bowel Management Difficulties
A range of difficulties associated with bowel management, ranging from ability to carry out their bowel program to worry about bowel accidents, performing their bowel program, and impact on everyday living
Pressure Ulcers
A range of difficulties associated with Pressure Ulcers, such as the extent to which pressure ulcers hinder engagement in social, cognitive, emotional, physical, and recreational activities
Pain Interference^†^
Consequences of pain on relevant aspects of one's life, including the extent to which pain hinders engagement with social, cognitive, emotional, physical, and recreational activities
Pain Behavior^†^
Self-reported external manifestations of pain; behavior, verbal or non-verbal and voluntary or deliberate, that typically indicate to others that an individual is experiencing pain, including observable displays, pain severity behaviors, and verbal reports of pain
Emotional Health
Positive Affect & Well-being*
Aspects of one's life that relate to a sense of well-being, life satisfaction or an overall sense of purpose and meaning
Depression*
Self-reported negative mood (sadness, guilt), views of self (self-criticism, worthlessness), and social cognition (loneliness, interpersonal alienation), as well as decreased positive affect and engagement (loss of interest, meaning, and purpose
Anxiety*
Symptoms that reflect autonomic arousal and experience of threat, including self-reported fear (fearfulness, panic), anxious misery (worry, dread), hyperarousal (tension, nervousness, restlessness), and somatic symptoms related to arousal (racing heart, dizziness)
Stigma^‡^
Others’ perceptions of oneself and publically enacted negativity, prejudice, and discrimination as a result of injury-related manifestations
Resilience
Subjective experience of the process and outcome of successfully adapting to difficult or challenging life experiences, especially highly stressful or traumatic events
Grief/Loss
Emotional reactions of grief (the natural process of reacting to a loss) such as anger, guilt, anxiety, sadness, and despair
Self-Evaluation
Assessment of one's emotional, evaluative, and cognitive perceptions of competence and worth
Psychological Trauma
An overwhelming experience of fear, helplessness or horror as a result of exposure to actual or perceived threat(s) to life, bodily integrity or the mind, usually rendering an individual unable to adequately cope
Social Participation
Ability to Participate in Social Roles and Activities*
Degree of involvement in one's usual social roles, activities, and responsibilities, including work, family, friends, and leisure
Satisfaction with Social Roles and Activities*
Satisfaction with involvement in one's usual social roles, activities, and responsibilities, including work, family, friends, and leisure
Independence/Autonomy
Perceived independence, ability to tell others about one's needs, and sense of control over one's life
Physical Functioning
Basic Mobility
Ability to carry out activities involving changing and maintaining body positions, transfers, moving and carrying objects, moving around in different locations
Self-Care
Ability to carry out activities involving eating, dressing, grooming, bathing and toileting, including managing bowel and bladder programs
Fine Motor Function
Ability to manually hold, manipulate and move objects that require varying degrees of dexterity and/or strength
Wheelchair Mobility
Ability to transfer in and out of a wheelchair, maneuver a wheelchair under different conditions, engage in activities from a wheelchair and manage wheelchair parts
Ambulation
Ability to engage in walking activities in different locations that vary based on speed, time and condition and the ability to manage stairs under different conditions

*Definition from Neuro-QOL.^35^

^†^Definition from PROMIS.^36^

^‡^Adapted from Neuro-QOL.^35^

Thirteen SCI-QOL item banks exhibited excellent fit statistics and have been developed as an IRT-calibrated item bank which may be administered as a full item bank, CAT, or short form (SF). Independence has a limited number of items (8 items) and a high RMSEA indicating possible multidimensionality but was also developed as an IRT-calibrated item bank. For three item pools, decreased sample sizes due to sparse cells in conjunction with a small number of acceptable items limited our ability to develop calibrated item banks which may be administered as CATs. Pressure Ulcers, Bladder Complications, and Pain Behavior are available as IRT-calibrated fixed-length scales.

All calibrated item banks demonstrated excellent internal consistency reliability. Coefficient alpha values ranged from 0.89 (Independence) to 0.97 (Positive Affect and Well Being). The three fixed-length scales demonstrated a high degree of internal consistency with coefficient alpha values ranging from 0.79 (Bladder Complications) to 0.92 (Pressure Ulcers).

Detail on the number of items tested, the number of items retained, and final CFA results are located in Table 6. Details on the individual iterations and reasons for item removal may be found in the individual domain-specific manuscripts throughout this special issue (e.g. Kisala *et al.*,^38^ Victorson *et al.*,^39^ Kalpakjian *et al.*).^40^ Bivariate correlations between Firestar-simulated CAT scores and SF scores on each domain can be found in Table 8.

**Table 8:** Correlations between Short Form and simulated CAT scores

		Calibration Data (CAT + SF simulations with full-bank data)	Reliability Study Data (CAT + SF administration with no duplicates)	
	*n*	Pearson's r	*n*	Pearson's *r*
Ambulation	825	0.98**	n/a	n/a
Ability to Participate	641	0.93**	164	0.92**
Anxiety	716	0.94**	175	0.93**
Basic Mobility	773	0.94**	85	0.95**
Bladder Management Difficulties	757	0.94**	168	0.94**
Bowel Management Difficulties	757	0.95**	169	0.95**
Depression	716	0.94**	175	0.95**
Fine Motor	849	0.95**	84	0.96**
Grief/Loss	716	0.97**	174	0.96**
Independence	629	0.99**	168	0.99**
Pain Interference	757	0.96**	171	0.96**
Positive Affect& Well-Being	716	0.95**	175	0.94**
Psychological Trauma	716	0.96**	89	0.95**
Resilience	716	0.96**	174	0.95**
Satisfaction with Social Roles & Activities	641	0.92**	166	0.91**
Self Care	788	0.94**	85	0.97**
Self-Esteem	716	0.92**	89	0.91**
Stigma	611	0.92**	175	0.91**
Wheelchair Mobility: *Power WC SF*	355	0.91**	45	0.91**
Wheelchair Mobility: *Manual WC SF*	435	0.89**	41	0.93**

**P<0.01.

Note: Ambulation items were not administered in the Reliability Study.

Seven of the developed item pools were not analyzed. Due to the low number of individuals in our calibration sample who had experienced respiratory complications (i.e. only 34% of the sample had ever experienced respiratory complications since their SCI, and the Respiratory items are in the context of the past 7 days), we lacked an adequate distribution of responses across cells to move forward with analysis of this subdomain. Further analysis and finalization of the Respiratory item pool will require an additional wave of data collection in a sample of individuals who have recently experienced respiratory complications as a result of their SCI. Similarly, many of the Sexual Functioning items required that the participant had a sexual partner (many of whom did not), and therefore, were not completed by a sufficient number of individuals to conduct IRT analyses. Finally, additional Control over Participation and Involvement in Life Situations item pools consisted primarily of items from the Community Participation Indicators^41^ measure and were not calibrated as part of this study.

## Phase V: Psychometric evaluation of final SCI-QOL CATs/SFs in a new sample

### Phase 5: Psychometric evaluation in new sample - METHODS

The overall purpose of this phase of research was to test the psychometric properties of the SCI-QOL measurement system in a new, independent sample. The SCI-QOL CATs and Short Forms were administered at an initial baseline interview (at study enrollment). A retest assessment was conducted between 7–14 days post baseline. All items were administered in interview format by trained examiners; this methodology helped ensure that the same individual with completing the assessment at both time points. Bivariate correlations (Pearson's *r*) and intraclass correlation coefficients (ICC) were computed to compare SCI-QOL CAT test-retest reliabilities. In general, the performance on the two administrations should be highly related with correlations coefficients >0.80. Test-retest reliability coefficients of a slightly lower magnitude (e.g. between 0.70 and 0.80) would be considered acceptable. Pearson's *r* correlation coefficients were also computed between the CAT and SF versions of each bank at the baseline assessment, to empirically test the assumption that SF and CAT scores will be nearly equal given the underlying IRT parameters.

#### Phase 5: Psychometric evaluation in new sample - Participants

A sample of 245 individuals with SCI was recruited from the following four SCI Model Systems (SCIMS) centers: University of Michigan, Kessler Foundation/Kessler Institute for Rehabilitation, Rehabilitation Institute of Chicago, and Craig Hospital. The reliability study was part of a larger research project wherein SCI-QOL CATs and static short forms were assessed serially at multiple intervals over a longer study period. The study protocol was reviewed and approved by each site's Institutional Review Board. Persons with a traumatic SCI that had been documented in their medical chart, who were 18 years or older, and who could read, speak, and understand English fluently were eligible to participate. The sample was stratified by level (paraplegia versus tetraplegia) and completeness of injury (complete vs. incomplete) to ensure that the final sample was a heterogeneous sample of individuals with SCI. All participants were community-dwelling individuals who were injured more than four months before the assessment and were stratified by diagnosis (paraplegia vs. tetraplegia), severity (complete vs. incomplete), and time since injury (≤2 years, >2 years). Each participant's diagnosis was confirmed by medical records and each participant's neurologic level was documented by their most recent American Spinal Injury Association Impairment Scale (AIS) rating.^24^

#### Phase 5: Psychometric evaluation in new sample - Data collection procedures

The CAT administration and all data collection were performed through a web-interface connected to the Assessment Center^SM^. All data points were obtained in a structured interview with a trained research assistant reading the questions from a computer screen and entering responses directly into the Assessment Center data platform. A detailed Manual of Procedures was also prepared and distributed to all sites. Throughout the data collection for validation study, data collectors from all sites participated in biweekly conference calls with the study coordinator to discuss progress and goals, specifically with regard to meeting sampling stratification goals.

## Phase 5: Psychometric evaluation in new sample - RESULTS

Values of Pearson's r coefficients indicate that scores on the baseline and retest assessments are highly related for each of the SCI-QOL banks and scales. As seen in Table 9, Pearson's *r* values for calibrated SCI-QOL item banks range from 0.74 (Bowel Management Difficulties) to 0.96 (Self Care). Pearson *r* values for SCI-QOL fixed length scales range from 0.70 (Bladder Complications) to 0.79 (Pressure Ulcers). Furthermore, ICC (2,1) values for item banks range from 0.74 (Ability to Participate; 95% CI: 0.67 to 0.79) to 0.96 (Self Care; 95% CI: 0.94 to 0.97). ICC (2,1) values for the fixed-length scales range from 0.69 (Bladder Complications; 95% CI: 0.61 to 0.76) and 0.79 (Pressure Ulcers; 95% CI: 0.74 to 0.84).

**Table 9:** SCI-QOL Test-Retest Reliability

SCI-QOL Item Banks	Pearson's *r*	Intraclass Correlation Coefficient	
		ICC (2,1)	95% CI
Ability to Participate in SRA	0.75**	0.74	(0.67, 0.79)
Anxiety	0.80**	0.80	(0.75, 0.84)
Basic Mobility	0.93**	0.93	(0.90, 0.94)
Bladder Management Difficulties	0.77**	0.76	(0.70, 0.81)
Bowel Management Difficulties	0.74**	0.74	(0.68, 0.79)
Depression	0.80**	0.80	(0.75, 0.84)
Fine Motor	0.95**	0.95	(0.93, 0.96)
Grief	0.84**	0.83	(0.78, 0.87)
Independence (*n* *=* 159)	0.84**	0.84	(0.78, 0.88)
Pain Interference (*n* *=* 244)	0.84**	0.83	(0.78, 0.87)
Positive Affect & Well Being	0.78**	0.78	(0.72, 0.82)
Resilience	0.79**	0.79	(0.74, 0.83)
Satisfaction with SRA	0.78**	0.77	(0.72, 0.82)
Self Care	0.96**	0.96	(0.94, 0.97)
Self Esteem	0.84**	0.84	(0.80, 0.88)
Stigma	0.80**	0.79	(0.74, 0.84)
Trauma	0.84**	0.84	(0.80, 0.88)
Wheelchair Mobility (*n* = 195)	0.92**	0.92	(0.90, 0.94)
SCI-QOL Scales			
Bladder Complications	0.70**	0.69	(0.61, 0.76)
Skin-Pressure Ulcers	0.79**	0.79	(0.74, 0.84)

**P < 0.01.

Note: Test-retest reliability was not assessed for Ambulation or Pain Behavior.

The simulated and empirically tested correlation coefficients between the CAT and SF administrations of the same bank are located in Table 8. CAT and SF scores were consistently very highly related. For CAT simulations from calibration data were compared to SF scores from the same dataset, Pearson's *r* values ranged from 0.92 (Satisfaction with SRA and Self Esteem) to 0.99 (Independence). When reliability study participants completed CATs and then subsequently completed any remaining items in the short form (i.e. ‘No Duplicates’ option in Assessment Center) with Pearson's *r* values ranging from 0.91 (Satisfaction with SRA, Self Esteem, Stigma, and Power Wheelchair Mobility) to 0.99 (Independence).

## Discussion

The SCI-QOL has been developed specifically with and for individuals with SCI. The SCI-QOL is innovative in its use of cutting edge qualitative and quantitative methods throughout its development and calibration. The SCI-QOL project team has consistently adhered to the scientific standards laid out by the PROMIS network,^1^ and has incorporated innovative psychometric techniques by transforming SCI-calibrated item banks back to the PROMIS or Neuro-QOL metrics.

There are PROMIS and Neuro-QOL scales that are intended to be appropriate across all health conditions, and the SCI-QOL has included many of these items verbatim. As such, this project marks the largest test of those PROMIS and Neuro-QOL banks in SCI to date. As described in later papers in this issue, some areas of these scales, such as anxiety and depression, are universally applicable; in these areas, with the SCI-QOL banks consisting of primarily of PROMIS items, but with updated IRT parameters to optimize measurement for individuals with SCI. In other areas, however, existing items were not as relevant in an SCI population. Within the Ability to Participate in Social Roles and Activities (SRA) and Satisfaction with SRA item banks, for example, there were many work-related items from PROMIS and Neuro-QOL that were psychometrically problematic due to their inapplicability (i.e. there were bimodal distributions due to the large number of individuals with SCI who are unable to return to work due to physical or financial reasons) due to their inapplicability. The SCI-QOL versions of these banks, therefore, are optimized not only in terms of administration order but also in the selection of items included in the final banks. One of the most important goals in developing the SCI-QOL system was to ensure that individuals with SCI are no longer faced with forms containing irrelevant (and potentially offensive) items when researchers or clinicians attempt to assess HRQOL.

Notably, though the SCI-QOL versions of existing banks have been optimized for SCI, the scores have been transformed to reflect the original metric (i.e. PROMIS or Neuro-QOL), thereby using SCI-specific development work and item calibrations, but yielding scores referencing the general population in order to ensure comparability across different studies and even across populations. In addition to the expanding these existing measurement systems, SCI-QOL has broken new ground by developing item banks in areas that are broadly relevant but deemed especially important in individuals with SCI (such as Resilience and Grief/Loss), as well as those that tend to be very specific to SCI, such as Bladder Management Difficulties, Bowel Management Difficulties, and Pressure Ulcers. Future directions include testing the Respiratory and Sexual Functioning items in larger samples of individuals for whom these items are directly relevant to ensure adequate distributions of responses to perform IRT analysis.

### Study limitations

Further work is needed on the responsiveness of this scale, in light of how individuals with SCI evolve and change following their injury. Results from ongoing intervention studies that administer SCI-QOL item banks both pre- and post-intervention will provide additional evidence for responsiveness of SCI-QOL measures to observed change in individuals with SCI. Establishment of clinically relevant scoring classifications and minimal clinically important differences will be important in facilitating use of SCI-QOL in clinical trials. Further, the research team must continue to marshal data in the process of gathering additional validation data on the SCI-QOL measurement system. Finally, stakeholder participation in all phases of the study has been limited to those individuals with traumatic SCI. It remains to be seen if results are generalizable to individuals with SCI of non-traumatic etiology.

## Conclusions

The SCI-QOL measurement system has been developed through multiple phases of research using advanced qualitative and quantitative methods, advanced computer technology and modern psychometric theory. Nineteen SCI-QOL item banks and 3 fixed scales, including those described in the 11 topic-specific manuscripts in this special issue as well as the physical functioning banks which have been described previously,^14,18,42^ have utilized the methodology described in this manuscript. All SCI-QOL instruments are available for SCI research or clinical practice through Assessment Center or via the lead author.

## Disclaimer statements

**Contributors** All authors have contributed significantly to the design, analysis and writing of this manuscript. The contents represent original work and have not been published elsewhere. No commercial party having a direct financial interest in the results of the research supporting this article has or will confer a benefit upon the authors or upon any organization with which the authors are associated.

**Funding** This study was supported by grant #5R01HD054659 from the National Institutes of Health – Eunice Kennedy Shriver National Institute of Child Health and Human Development/National Center on Medical Rehabilitation Research and the National Institute on Neurological Disorders and Stroke. The SCI-FI development work was supported by the US Department of Education, National Institute of Disability and Rehabilitation Research Grant Numbers: H133N120002, H133N060014, H133N110006, H133N110020, H133N110002, H133N110021, H133N110011, H133N110007, H133N110009.

**Conflicts of interest** All SCI-QOL Items are copyright © 2015 David Tulsky and Kessler Foundation. All rights reserved. All SCI-QOL items originally from Neuro-QOL are copyright © 2008-2013 David Cella on behalf of the National Institute for Neurological Disorders and Stroke (NINDS). All items are freely available to the public via the Assessment Center platform (http:\\www.assessmentcenter.net) and, at present, there are currently no plans for Dr. Tulsky, Kessler Foundation, or the NINDS to profit from the use of the copyrighted material.

**Ethics approval** Institutional Review Board approval was received at each participating site.
